# Proteomic Analysis Identified DJ-1 as a Cisplatin Resistant Marker in Non-Small Cell Lung Cancer

**DOI:** 10.3390/ijms12063489

**Published:** 2011-06-01

**Authors:** Hua-Zong Zeng, Yi-Qing Qu, Wen-Jun Zhang, Bing Xiu, An-Mei Deng, Ai-Bin Liang

**Affiliations:** 1 School of Life Sciences and Technology, Tongji University, Shanghai 200092, China; E-Mail: zeng@tongji.edu.cn; 2 Department of Respiratory Medicine, Qilu Hospital, Shandong University, Jinan 250012, China; E-Mail: yiqingqu@126.com; 3 Central Laboratory, Tongji Hospital of Tongji University, Tongji University, Shanghai 200065, China; E-Mails: lab7182@126.com (W.-J.Z.); lab7182@126.com (B.X.); 4 Laboratory Diagnostics, Shanghai Changhai Hospital, Shanghai 200003, China

**Keywords:** non-small lung cancer, cisplatin, resistance, DJ-1, proteomics

## Abstract

The aim of study is to identify cisplatin-resistance associated biomarkers for non-small cell lung cancers (NSCLC). We use two-dimensional electrophoresis (2-DE) combined with MALDI-TOF mass spectrometry to compare the proteome between lung cancer cell line A549 and its cisplatin-resistant subline A549/DDP. Nine cisplatin resistance-related proteins were identified, and DJ-1, one of the differently expressed proteins, was selected for further validation and evaluation. Immunohistochemical results demonstrated that high expression level of DJ-1 was associated with cisplatin resistance and a predictor for poor prognosis in 67 locally advanced NSCLC patients. Furthermore, *in vitro* results showed that silencing DJ-1 increased the proliferation inhibitory effect of cisplatin to A549/DDP cells. In conclusion, DJ-1 might play an important role in the resistibility to cisplatin, and it could also act as a novel candidate biomarker for predicting the response of NSCLC patients to cisplatin-based chemotherapy.

## 1. Introduction

Non-small cell lung cancer (NSCLC) accounts for most lung cancer cases. Currently, more than half of the patients diagnosed with NSCLC were in their advanced stages at diagnosis [1]. Cisplatin-based combination chemotherapies are used as standard treatment modalities for advanced NSCLC patients with good performance status, and have shown a significant improvement in overall survival and quality of life. However, there are still some patients who exhibit drug resistance to cisplatin-based chemotherapy [2]. Therefore, identifying novel molecular markers related to cisplatin resistance would be helpful to further understand the exact mechanisms of chemo-resistance and make more individual chemotherapy strategies for NSCLC patients in the future.

Previous studies have identified multiple genetic aberrations that might contribute to cisplatin resistance in NSCLC patients. For example, Cobo *et al*. [3] reported that excision repair cross-complementing 1 (ERCC1) mRNA expression confers selective resistance to cisplatin in NSCLC patients. Kim *et al*. [4] revealed that the BRCA1 haplotype is significantly associated with survival of NSCLC patients treated with platinum-based chemotherapy. Recently, it was also suggested that transglutaminase 2 (TGM2) is a cisplatin resistance marker in non-small cell lung cancer [5]. With these drug resistance molecules, much effort is still needed to find novel biomarkers with potential utility in predicting the cisplatin resistance of NSCLC patients.

In this present study, we first performed a proteomic approach to compare the differently regulated protein expression profiles between cisplatin-sensitive and -resistant lung cancer cell lines to screen candidate molecules related to cisplatin chemoresistance. Then we further confirmed that DJ-1, one of the differently regulated marker identified in proteomic analysis, is a novel cisplatin-resistant marker and an independent prognostic factor for locally advanced NSCLC patients. In addition, we also validated an association between silencing DJ-1 with decreased cisplatin resistance *in vitro*.

## 2. Results and Discussion

### 2.1. Proteomic Analysis Identified DJ-1 Up-Regulated in Drug-Resistant A549/DDP Compared with Drug-Sensitive A549 Cells

**Figure 1:**
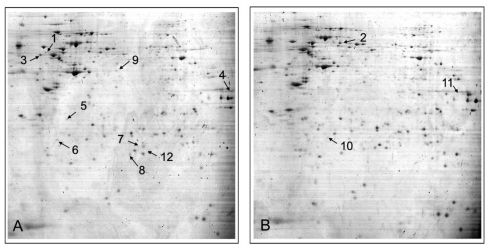
Representative two-dimensional electrophoresis gels (pI 3–10 NL, 18 cm) showing that 9 protein spots up-regulated in A549/DDP cells (**A**) and 3 protein spots down-regulated compared with A549 cells (**B**) with arrows.

**Figure 2:**
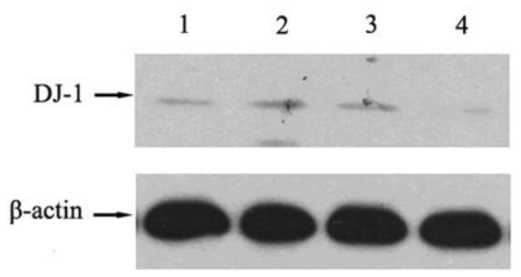
Western-blot results showed that cisplatin-resistant A549/DDP cells (**2**) had a higher DJ-1 expression than A549 cell (**1**); siRNA transfected A549/DDP cells (**4**); had a decreased DJ-1 expression level than mock RNAi transfected A549/DDP cells (3).

In this present study, we adopted a 2-DE analysis to quantitatively compare the protein profilings of NSCLC cell line A549 and its cisplatin-resistant subline A549/DDP. The differently expressed proteins with more than 2 fold changes between the two groups were selected to perform protein identification by MALDI-TOF. A total of 12 proteins were successfully identified, including 9 up-regulated proteins and 3 down-regulated proteins (Figure 1 and Table 1). The different expression of DJ-1 between A549 and A549/DDP cells identified in proteomic study was further validated by Western blot analysis. As shown in Figure 2, A549/DDP cells had an increased expression level of DJ-1 compared to A549 cells, which is consistent with the finding of the proteomic analysis.

### 2.2. DJ-1 Correlated to Cisplatin Resistance and Predicted Poor Prognosis in Locally Advanced NSCLC Patients

**Figure 3:**
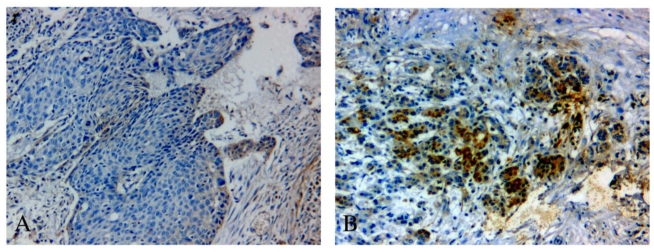
Representative images of immunostaining of DJ-1 in primary advanced non-small cell lung cancer tumors with DJ-1-low expression (**A**) and DJ-1-high expression (**B**) (200× magnification).

To evaluate the clinicopathological significance of DJ-1 expression in NSCLC patients, we performed an immunohistochemical analysis on 67 locally advanced NSCLC tumor tissues. DJ-1 staining mainly located in the cytoplasm of cancer cells in NSCLC tissues (Figure 3). NSCLC cases were categorized into two groups based on the median H score: DJ-1-high group (*n* = 33, with an *H* score >28%) and DJ-1-low group (*n* = 34, with an *H* score ≤28%). As seen in Table 2, DJ-1-high group had a significantly higher frequency of cisplatin resistance than DJ-1-low group (57.6% *vs*. 29.4%, *P* = 0.020). No significant association was identified between DJ-1 expression and other clinicopathological characteristics.

**Figure 4:**
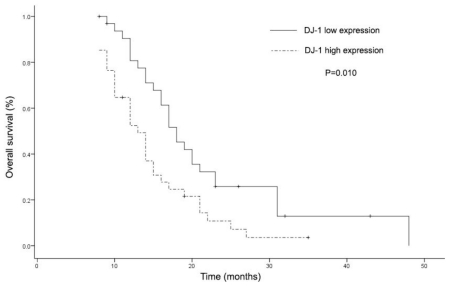
Kaplan-Meier survival curves for overall survival (OS) of patients with locally advanced non-small cell lung cancer according to the expression level of DJ-1 (log rank test, *P* = 0.010).

When the overall survival analysis of these patients was performed according to DJ-1 expression level and other clinicopathological characteristics, cases with high DJ-1 expression level had a significantly shorter overall survival time compared with DJ-1-low subgroup (Figure 4, Table 3). Multivariate analysis was not performed because there was no other variable that might be considered in relation to overall time.

### 2.3. Silencing of DJ-1 Partially Reversed Cisplatin Resistance in Drug-Resistant A549/DDP Cells

**Figure 5:**
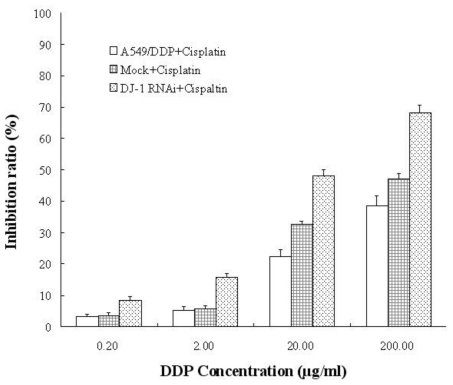
The relationship between silencing of DJ-1 and cisplatin resistance reversion in A549/DDP cells. After treated with increasing concentration of cisplatin, siRNA targeting DJ-1 transfected A549/DDP cells had a higher inhibition rate than the mock-transfected and untransfected controls (*P* < 0.01, ANOVA test).

To explore the relationship between DJ-1 expression and cisplatin-resistance *in vitro*, we performed siRNA treatment to down-regulate the DJ-1 expression level in A549/DDP cells (Figure 2), and then treated increasing concentrations of cisplatin. As seen in Figure 5, when the DJ-1 level was decreased in resistant lung cancer cells, the sensitivity to cisplatin increased. CKK-8 assay results showed that siRNA targeting transfected lung cancer cells had a higher inhibitory ratio than mock RNAi transfected controls after treatment with increasing concentrations of cisplatin.

### 2.4. Discussion

Mining novel genes or proteins associated with cisplatin-resistance could not only be helpful in revealing the mechanisms underlying cisplatin resistance, but also be of potential utility in the prediction of cisplatin resistance for NSCLC patients. The recently developed proteomic techniques provide an excellent tool in identifying novel cisplatin-resistance markers. For example, Gong *et al*. [6] identified 10 unique proteins differently expressed between cisplatin-sensitive and -resistant ovarian cancer cell lines by 2-DE and ESI-Q-TOF MS/MS analysis. Li *et al*. [7] employed the iTRAQ-LC-MSMS proteomic approach to compare the protein profilings between cisplatin-resistant COC1/DDP cell line and its parental COC1 cell line, and validated that PKM2 and HSPD1 could contribute considerably to the cisplatin resistance in ovarian cancer cells.

In this study, we performed a 2-DE combined with MALDI-TOF analysis to identify differential proteins expression between cisplatin-sensitive (A549) and cisplatin-resistant (A549/DDP) lung cancer cell lines. We successfully identified 12 unique proteins with significantly altered expression levels. Based on their functions, these proteins are mainly involved in protein binding and cytoskeletal proteins. Some of these proteins such as phosphoglycerate kinase 1 (PGK1), vimentin (VIM) and stathmin (STMN1), have been reported to be associated with chemotherapy resistance in previous proteomic studies, while our study also found several novel candidate resistance-associated proteins such as DJ-1, which will be valuable for further study of the mechanisms of cisplatin resistance and the clinical utility as a resistance marker.

DJ-1 encodes a highly conserved protein belonging to the Thij/PfpI/DJ-1 superfamily [8]. With respect to cancer, increasing evidence suggests that DJ-1 plays an important role in tumor progression as a potent activator of the PI3K/Akt, mTOR and HIF1 pathway, and an inhibitor of p53-mediated apoptosis [8–10]. DJ-1 is frequently overexpressed in a large variety of solid tumors. Most recently, Kim *et al*. [11] identified DJ-1 expression increased in 19 of 23 primary non-small cell lung carcinoma samples compared to paired nonneoplastic lung tissues. However, until now, no previous study has reported the association between DJ-1 expression with cisplatin resistance, and the clinicopathological significance of DJ-1 in NSCLC is also unknown. Therefore, we selected DJ-1 as a cisplatin-resistance tumor marker for further validation.

Our Western blot results validated that DJ-1 expression increased in cisplatin-resistant lung cancer cells compared with its sensitive parent cell. In the following immunohistochemistry study, we found that DJ-1 expression level is positively associated with cisplatin resistance, and is also a prognostic factor for shorter survival time in NSCLC. Furthermore, in *in vitro* experiment, we also demonstrated that down-regulation of DJ-1 by RNAi in cisplatin-resistant lung cancer cells could sensitize them to cisplatin. These findings suggest that DJ-1 might participate in cisplatin resistance in NSCLC, and could serve as a potential biomarker for resistance prediction and prognosis.

Considering that many previous studies have proved that PI3K/Akt, mTOR and HIF1 pathways have been confirmed to be involved in the cisplatin-resistance of cancer cells [12–15], we suggest that DJ-1 might mediate cisplatin resistance in NSCLC as an activator of these pathways, however, further works need to be done to clarify its definite function in chemotherapy resistance.

## 3. Experimental Section

### 3.1. Cell Lines and Patients

Human lung adenocarcinoma cell line A549 cells were obtained from the American Type Culture Collection (ATCC), and its cisplatin-resistant subline A549/DDP was obtained from XiangYa Cell center, Changsha, China. Cells were maintained in RPMI-1640 medium supplemented with 10% heat-inactivated fetal bovine serum, for A549/DDP cells, 2 μg/mL cisplatin was added. Three replicate tests were performed for all the *in vitro* analysis.

A total of 67 locally advanced NSCLC patients including 37 squamous cell carcinomas and 30 adenocarcinomas undergoing resection or biopsy in Shandong University Medical College (Jinan, China) and Tongji Hospital (Shanghai, China) between February 2000 and July 2007 were included in this study. All the patients included were treated with at least three cycles of post-operational cisplatin-based third-generation chemotherapy doublets including NP regimens (NVB and DDP), TP regimens (Taxol and DDP), and GP regimens (Gemzar and DDP). Cisplatin-based chemotherapy responses were evaluated according to the WHO criteria, which classified the responses into complete response (CR), partial response (PR), stable disease (SD), and progressive disease (PD). Patients with CR and PR were defined as sensitive to cisplatin-based chemotherapy; the other patients with PR and SD were defined as resistant. The study was approved by the local ethics committees. All tumor specimens analyzed were collected before chemotherapy with informed consent. The detailed clinicopathological characteristics of the subjects are listed in Table 2.

### 3.2. Two-Dimensional Gel Electrophoresis (2-DE) and Mass Spectrometry Analysis

The A549 cells and A549/DDP cells were harvested and lysed in a buffer containing 7 M urea, 2 M thiourea, 4% w/v, 40 mM DTT, 0.5% IPG buffer (pH 3–10, NL). The lysate was then subjected to centrifugation at 10,000 rpm for 10 min to remove cell debris. Bradford assay was used to determine the total protein concentration. 500 μg of protein samples diluted in lysis buffer were applied to 18 cm non-linear IPG strips pH 3–10 (Bio-Rad). IEF were performed in a Protean IEF cell apparatus (Bio-Rad). After the first dimension, the strips were balanced in an equilibration buffer (6 M urea, 75 mM Tris-HCl pH 8.8, 29.3% glycerol, 2% SDS and 0.002% bromophenol blue) containing 1% DTT for 10 min, and then in the same equilibration buffer containing 2.5% iodoacetamide instead of DTT for an additional 10 min. The second dimension electrophoresis was carried out using Protean II XL cell apparatus (Bio-Rad). The protein gels were visualized by Coomassie Brilliant Blue R350 (Amersham Biosciences), and scanned with a GS-800 Calibrated Densitometer (Bio-Rad) at 300 dpi resolution. Images were analyzed using PDQuest software (Bio-Rad). Differently expressed spots were excised from 2-DE gels and in-gel digested with modified trypsin (Promega). Extracted peptides were analyzed by a Voyager-DE STR MALDI-TOF mass spectrometer (Applied Biosystems). Database searching of MS data was performed using Mascot software (Matrix Science Ltd).

### 3.3. Western-Blot

About 30 μg extracted protein samples were separated by 12% SDS-polyacrylamide gel electrophoresis, and then nitrocellulose membrane transferring was conducted. The membrane was blocked with 5% defatted milk and incubated with a rabbit polyclonal antibody against human DJ-1 (Abcam) and β-actin antibody (Santa Cruz Biotech) at 1:1000 dilutions overnight at 4 °C. Detection was performed using an ECL chemiluminescence reagent (Amersham).

### 3.4. Immunohistochemistry

Immunohistochemical staining was performed on 4 μm of formalin-fixed, paraffin-embedded sections of 67 NSCLC tumor tissues. Briefly, antigen retrieval was performed in 0.01 M citrate buffer (pH 6.0); endogenous peroxidase activity was blocked with hydrogen peroxide for 5 min at room temperature. Then sections were incubated with the same antibody against human DJ-1 used in Western-blot analysis (1:200 dilution) at 4 °C overnight. Visualization was performed using a DAKO EnVision system. Nonspecific rabbit IgG was used as a negative control.

Expression of DJ-1 stains were scored based on the percentage of positive tumor cells and the intensity of staining (graded on a scale of 0–3:0, no staining; 1, weak; 2, moderate; 3, strong). A semiquantitative H-score was obtained by multiplying the density and intensity of staining. Cases were categorized into two groups based on the median value of all the H-scores in NSCLC specimens: DJ-1-high (H-scores more than median value) and DJ-1-low (H-scores less than or equal to median value) [16].

### 3.5. RNA Interference and Cisplatin Sensitivity Assay

To silence DJ-1 expression, a small interfering RNA (siRNA) duplex targeting human DJ-1 (5′-GATTAAGGTCACCG-3′) was introduced into 1 × 10^6^ A549/DDP cells using lipofectamine (Invitrogen) according to the manufacturer’s instruction as described previously. Cells were double-transfected at 72 h intervals and then were harvested for further analysis. Specific silencing of the targeted gene was validated by Western blot.

After the transfection, 1 × 10^4^ A549/DDP cells were plated in 96-well plates and then were exposed to various concentrations of cisplatin (0.2–200 μg/mL) for 24 h. Following cisplatin treatment, the 10 μL of Cell Counting Kit-8 solution was added to each well and the mixture was incubated at room temperature for 1h. After the incubation, the absorbance at 450 nm was determined. The cell inhibition ratio was calculated as a fraction of the untreated controls.

### 3.6. Statistical Analysis

The correlation between DJ-1 expression and the clinicopathological characteristics of NSCLC were analyzed using Fisher’s exact test. The inhibition rates among groups were analyzed by ANOVA test. The overall survival (OS) time was defined as the time from operation to cancer-caused death. The survival curves were estimated by the Kaplan-Meier method, and differences were compared by the log-rank test. Statistically significant prognostic factors identified in univariate analyses were selected to enter multivariable analyses using a Cox proportional hazards model. Statistical analyses were performed using SPSS 11.0 software for Windows. A p value less than 0.05 was set as statistically significant.

## 4. Conclusions

In conclusion, in this present study, using a proteomic approach, we identified and validated DJ-1 being a novel cisplatin-resistance marker and a prognostic factor for advanced NSCLC patients. Furthermore, our *in vitro* demonstrated that inhibition of DJ-1 may overcome cisplatin resistance, which suggesting DJ-1 might serve as a potential target for the reversal of drug resistance in NSCLC patients, and ultimately lead to improved clinical outcomes.

## Figures and Tables

**Table 1:** Differently expressed proteins between A549/DDP and A549 lung cancer cells in proteomic analysis.

Spot	Gene Symbol	Protein Name	Swiss-Prot Accession	Fold *	Function
1	VIM	Vimentin	P08670	3.76	structural constituent of cytoskeleton protein C-terminus binding
2	ALDOA	Fructose-bisphosphate aldolase A	P04075	−3.09	actin binding fructose binding fructose-bisphosphate aldolase activity identical protein binding tubulin binding
3	HNRNPA2B1	Heterogeneous nuclear ribonucleoproteins A2/B1	P22626	2.82	RNA binding nucleotide binding protein binding single-stranded telomeric DNA binding
4	PGK1	Phosphoglycerate kinase 1	P00558	4.47	ATP binding phosphoglycerate kinase activity
5	PRDX4	Peroxiredoxin 4	Q13162	8.11	thioredoxin peroxidase activity
6	ANXA1	Annexin A2	P04083	5.93	calcium ion binding calcium-dependent phospholipid binding phospholipase A2 inhibitor activity protein binding, bridging receptor binding structural molecule activity
7	PARK7	Protein DJ-1	Q99497	5.43	protein binding
8	STMN1	Stathmin	P16949	3.49	signal transducer activity tubulin binding
9	PFN1	Profilin-1	P07737	2.95	actin binding proline-rich region binding
10	P07437	Tubulin β chain	TUBB	−5.31	GTP binding GTPase activity MHC class I protein binding
11	P09972	Fructose-bisphosphate aldolase C	ALDOC	−2.37	cytoskeletal protein binding fructose-bisphosphate aldolase activity
12	P23528	Cofilin-1	CFL1	3.94	actin binding

* A549/DDP *vs.* A549.

**Table 2:** Association of DJ-1 expression and clinicopathological characteristics in 67 locally advanced NSCLC patients.

Characteristics	Total (*n* = 67)	DJ-1 Low Expression (*n* = 34)	DJ-1 High Expression (*n* = 33)	*P* Value
**Gender**				
Male	48	26	22	0.373
Female	19	8	11	
**Age**				
<60 year	29	17	12	0.260
≥60 year	38	17	21	
**Histology**				
Squamous cell carcinoma	37	18	19	0.703
Adenocarcinoma	30	16	14	
**Pathologic stage**				
IIIA	9	3	6	0.261
IIIB	56	31	27	
**Chemotherapy regimens**				
NVB + DDP	29	17	12	
Taxol + DDP	23	10	13	0.521
Gemzar + DDP	15	7	8	
**Response to cisplatin-based chemotherapy**				
Resistant	29	10	19	0.020
Sensitive	38	24	14	

**Table 3:** Univariate analysis of overall survival with regard to clinicopathological characteristics.

Characteristics	Univariate Analysis	
	*HR* (95% CI)	*P*
Age	1.082 (0.641–1.825)	0.768
Gender	1.434 (0.800–2.571)	0.226
Histology	0.922 (0.547–1.556)	0.762
Pathologic stage	0.691 (0.313–1.525)	0.360
Chemotherapy regimens	0.609 (0.611–2.322)	0.609
Response to cisplatin-based chemotherapy	0.688 (0.403–1.174)	0.170
DJ-1 expression level	0.495 (0.291–0.843)	0.010
